# Supplementary material for: Quality of life of patients with solid malignancies at 3 months after unplanned admission in the intensive care unit: A prospective case-control study
Source: PLoS One. 2023 Jan 5;18(1):e0280027. doi: 10.1371/journal.pone.0280027 (PMC9815568; doi:10.1371/journal.pone.0280027)
Supplement: Attachment — Submitted filename: Reply to the editor_PlosOne final version.docx [file pone.0280027.s001.docx]

REPLY TO THE EDITOR:

28th November 2022

Dear Editor,

We would like to express our full appreciation for giving us the opportunity to revise our manuscript Ref. PONE-D-22-19984. We believe that the reviewers made insightful suggestions that greatly helped to improve the manuscript. In red you will find the changes taking into account remarks of reviewers and highlighted in yellow the corrected English

We expect that this manuscript can be now suitable for publication in *PLOS One*. We remain at your disposal to clarify any pending point.

Yours sincerely,

Wassila Marnas, MD

Anne-Claire Toffart, MD, PhD

**Answers to Journal Requirements**

When submitting your revision, we need you to address these additional requirements.

1. Please ensure that your manuscript meets PLOS ONE's style requirements, including those for file naming. The PLOS ONE style templates can be found at

<https://journals.plos.org/plosone/s/file?id=wjVg/PLOSOne_formatting_sample_main_body.pdf> and

<https://journals.plos.org/plosone/s/file?id=ba62/PLOSOne_formatting_sample_title_authors_affiliations.pdf>

R : Done

2. PLOS requires an ORCID iD for the corresponding author in Editorial Manager on papers submitted after December 6th, 2016. Please ensure that you have an ORCID iD and that it is validated in Editorial Manager. To do this, go to ‘Update my Information’ (in the upper left-hand corner of the main menu), and click on the Fetch/Validate link next to the ORCID field. This will take you to the ORCID site and allow you to create a new iD or authenticate a pre-existing iD in Editorial Manager. Please see the following video for instructions on linking an ORCID iD to your Editorial Manager account: <https://www.youtube.com/watch?v=_xcclfuvtxQ>.

R : https://orcid.org/0000-0003-4377-0227

3. Please amend the manuscript submission data (via Edit Submission) to include author “W M’Sallaoui”

R : W M’Sallaoui is Wassila Marnas. We modified in the Edit Submission Marnas in M’Sallaoui

4. Please amend your authorship list in your manuscript file to include author “Juliette Piot, Wassila Marnas”

R : Wassila Marnas is W M’Sallaoui. Juliette Piot is still in our authorship list. We modified tis authorship list as requested by Plos One.

5. We note that you have indicated that data from this study are available upon request. PLOS only allows data to be available upon request if there are legal or ethical restrictions on sharing data publicly. For more information on unacceptable data access restrictions, please see <http://journals.plos.org/plosone/s/data-availability#loc-unacceptable-data-access-restrictions>.

In your revised cover letter, please address the following prompts:

a) If there are ethical or legal restrictions on sharing a de-identified data set, please explain them in detail (e.g., data contain potentially sensitive information, data are owned by a third-party organization, etc.) and who has imposed them (e.g., an ethics committee). Please also provide contact information for a data access committee, ethics committee, or other institutional body to which data requests may be sent.

b) If there are no restrictions, please upload the minimal anonymized data set necessary to replicate your study findings as either Supporting Information files or to a stable, public repository and provide us with the relevant URLs, DOIs, or accession numbers. For a list of acceptable repositories, please see <http://journals.plos.org/plosone/s/data-availability#loc-recommended-repositories>.

We will update your Data Availability statement on your behalf to reflect the information you provide

R : Data cannot be shared publicly because of contains potentially identifying or sensitive patient information. Data are available from the Grenoble Alpes Universityt Hospital Institutional Data Access (contact via DRCI@chu-grenoble.fr) for researchers who meet the criteria for access to confidential data.

6. Please review your reference list to ensure that it is complete and correct. If you have cited papers that have been retracted, please include the rationale for doing so in the manuscript text, or remove these references and replace them with relevant current references. Any changes to the reference list should be mentioned in the rebuttal letter that accompanies your revised manuscript. If you need to cite a retracted article, indicate the article’s retracted status in the References list and also include a citation and full reference for the retraction notice.
R : Done. We have not modified our reference list.

## **Answers to Reviewers’ Comments:**

4. Is the manuscript presented in an intelligible fashion and written in standard English?

PLOS ONE does not copyedit accepted manuscripts, so the language in submitted articles must be clear, correct, and unambiguous. Any typographical or grammatical errors should be corrected at revision, so please note any specific errors here.

Reviewer #1: No

Reviewer #2: Yes

R : Done, we reviewed English.

Reviewer #1:

Methods:
I wonder if there are pre-existing criteria for admission to the ICU at the authors’ organization, e.g. based on performance status; co-morbidities; use of TLT etc. If so, it would be helpful to describe these in this section.

R : The decision to admit to ICU is based on the criteria of each patient, taking into account known prognostic factors. There are no standardized pre-existing criteria.

We added these sentences in the manuscript (page 6, §2)

How were patients identified? At the time of admission to the ICU, or after discharge?

R : Patients were identified at ICU discharge.

We added “Patients were identified at ICU discharge, and included between 5 and 14 days after ICU discharge.” (page 6, §2)

How soon afterwards were they approached about the study?

R : Patients were included between 5 and 14 days after ICU discharge.

We added : “Patients were identified at ICU discharge, and included between 5 and 14 days after ICU discharge.” (page 6, §2)

Who completed the SF-36, patients or their family members? If the latter, this should be described in the paper.

R : The SF-36 was completed by the patients during a telephone interview

We added : « which the patients answered during a telephone interview. » (page 8, §2)

Can the authors speak to their inclusion criteria of lung, colorectal and head and neck cancers only?

R : Based on a previous publication “Gheerbrant H, Timsit JF, Terzi N, Ruckly S, Laramas M, Levra MG, et al. Factors associated with survival of patients with solid Cancer alive after intensive care unit discharge between 2005 and 2013. BMC Cancer. 5 janv 2021;21(1):9.”, we identified lung, colorectal and head and neck cancers as the most frequent cancers admitted in our ICUs.

We added: “Adult patients were included if they had lung, colorectal, or head and neck cancer (the three most common solid tumors encountered at the ICU) [27] “(page 6, §2)

Several other studies have shown that social supports influenced QOL after an ICU stay. Did the authors consider including this as a potential factor for this study?

R : We agree. Unfortunately, we did not collect data on social conditions. As far as French law is concerned, the study would be more complicated to carry out.

Results:
How many patients with cancer were admitted to the ICU during the study period (i.e. how many died in the ICU)?

R : We did not collect this data because that was beyond the objectives of this study. In our institution, between 2005 and 2013, 30% of patients (108/361) with solid tumors died in ICU (Gheerbrant et al. *BMC Cancer* 2021).

Only a small number of patients had colorectal or head and neck cancers. Were these less likely to be admitted to the ICU in the first place?

R : We cannot answer to this question. The location of cancer is not a variable used in the decision of admission to the ICU. In the literature, cancer patients in the ICU were described with hematological malignancies, localized or metastatic solid tumor, not regarding the location of cancer.

Consider including percentages in the tables for clarity.
I think there is an error in Table 1, where non-metastatic disease adds up to 35. I think it should be 34 (35/69 had metastatic disease according to the text)?

R : We thank you for this comment. We modified the Table 1.

Include an explanation for the IGS2 and SOFA scores for readers who may not be familiar with these.

R : Thank you for your comment: IGS2 is a French abbreviation. We modified in SAPS II. SAPS II and SOFA scores were usually used to described severity of patients admitted in ICU

We added « Severity scores in ICU were recorded: Simplified Acute Physiology Score II (SAPSII) [29] and Sepsis-related Organ Failure Assessment (SOFA) scores [30] » (page 8, §1)

There is a typo in Table 3. It says ‘change in eight’ instead of ‘change in weight’.

R : You are right. Thank you for this comment. We modified the Table 3.

Discussion:
‘These findings can be explained by the psychological care these patients received after a stay in the ICU’…can the authors elaborate on exactly what support these patients received?

R : That is an hypothesis. We did not collect data on the psychological follow-up of patients.

‘The incidence of severe infections acquired during the ICU stay was found [to be] higher in this population compared to non-cancer patients’. This sentence is unclear. Does it refer to the current study, in which case this information might be better presented in the results section? Alternatively, does it refer to patients with cancer admitted to the ICU in general, in which case it needs a reference?

R : This sentence does not refer to the current study. Here is the reference that was included in the article : Vincent JL, Rello J, Marshall J, Silva E, Anzueto A, Martin CD, Moreno R, Lipman J, Gomersall C, Sakr Y, Reinhart K; EPIC II Group of Investigators. International study of the prevalence and outcomes of infection in intensive care units. JAMA. 2009 Dec 2;302(21):2323-9. doi: 10.1001/jama.2009.1754. PMID: 19952319.

General comments

The small sample size and heterogeneity in terms of cancer diagnoses and stage of illness makes the findings difficult to interpret or generalize.

R : We agree. This point has been added in the limits of the study.

The absence of baseline QOL scores is also a challenge, in that the ICU group may have had higher QOL scores to begin with.

R : We agree. As mentioned in the discussion, assessing QOL at baseline, i.e. prior to ICU admission or equivalent, was not possible.

Additional demographic factors such as marital status, social supports, symptom burden might be useful in future studies exploring QOL.

R : We agree. According to the French law, collecting “marital status, social supports” are considered sensitive data. Collecting such data would have required a strong justification. This point has been mentioned in the Discussion.

There are quite a few grammatical errors throughout the paper. If considered for publication, I would recommend a native English speaker familiar with medical writing review it.

R : Done

Reviewer #2: I congratulate the authors for the well-designed and written manuscript that presents important contributions to the care of cancer patients admitted to the ICU. The limitations were well described and the conclusion adequately responds to the objectives.

R : We thank you for this positive comment.
